# Current Structural Knowledge on the CNNM Family of Magnesium Transport Mediators

**DOI:** 10.3390/ijms20051135

**Published:** 2019-03-06

**Authors:** Paula Giménez-Mascarell, Irene González-Recio, Cármen Fernández-Rodríguez, Iker Oyenarte, Dominik Müller, María Luz Martínez-Chantar, Luis Alfonso Martínez-Cruz

**Affiliations:** 1 Center for Cooperative Research in Biosciences (CIC bioGUNE), Bizkaia Science and Technology Park Bld 800, 48160 Derio, Bizkaia, Spain; pgimenez@cicbiogune.es (P.G.-M.); irecio@cicbiogune.es (I.G.-R.); cfernandez@cicbiogune.es (C.F.-R.); ioyenarte@cicbiogune.es (I.O.); mlmartinez@cicbiogune.es (M.L.M.-C.); 2 Department of Pediatric Gastroenterology, Nephrology and Metabolic Disorders, Charité Universitäts Medizin, Berlin, 13353 Berlin, Germany; Dominik.Mueller@charite.de; 3 Liver Disease Lab, CIC bioGUNE, Centro de Investigación Biomédica en Red de Enfermedades Hepáticas y Digestivas (CIBERehd), 48160 Derio, Bizkaia, Spain

**Keywords:** CNNM, ACDP, magnesium homeostasis, magnesium transport, CBS domain, cNMP domain, CNBH domain, Jalili syndrome, hypomagnesemia, cancer

## Abstract

The cyclin and cystathionine β-synthase (CBS) domain magnesium transport mediators, CNNMs, are key players in maintaining the homeostasis of magnesium in different organs. The human family includes four members, whose impaired activity causes diseases such as Jalili Syndrome or Familial Hypomagnesemia, but is also linked to neuropathologic disorders, altered blood pressure, and infertility. Recent findings demonstrated that CNNMs are associated with the highly oncogenic phosphatases of the regenerating liver to promote tumor growth and metastasis, which has attracted renewed focus on their potential exploitation as targets for cancer treatment. However, the exact function of CNNMs remains unclear and is subject to debate, proposed as either direct transporters, sensors, or homeostatic factors. This review gathers the current structural knowledge on the CNNM family, highlighting similarities and differences with the closely related structural partners such as the bacterial Mg^2+^/Co^2+^ efflux protein CorC and the Mg^2+^ channel MgtE.

## 1. Introduction

Magnesium (Mg) is an essential element that acts as a cofactor in hundreds of enzymatic reactions involved in the synthesis, folding, and stability of small and large biomolecules [1]. Mg also plays a key role in the stabilization of cell membranes, neuronal transmission [2], cardiac excitability, regulation of blood pressure, maintenance of nerve and muscle functions, and in the regulation of the glucose and insulin metabolism [3,4]. In its ionic form (Mg^2+^), magnesium antagonizes calcium (Ca^2+^) and functions as a signal transducer [5,6]. Human adults contain approximately 25 g of magnesium that is unequally distributed across different tissues. The largest amount is found in the bones (≈60%), where it resides on the surface of hydroxyapatite and in the hydration shell around the crystal, thus contributing to maintaining the integrity of the skeleton [7]. The remaining amount is localized in the muscles and soft tissues [8]. Only 1% of the total magnesium is in the blood. Considering its biological relevance, our body relies on complex molecular systems that ensure the bioavailability of this cation by establishing a careful balance between its absorption and excretion [9]. These processes were recently reviewed in detail [8,10]. However, the exact mechanisms by which the different channels, transporters, and sensors facilitate the passing of Mg^2+^ through the cell membranes remains poorly understood [11]. It is, however, well-established that loss-of-function alterations in Mg^2+^ transport machinery due, for example, to the presence of mutations cause a steady decrease in its serum and intracellular concentrations, which impacts the overall cell and organism functioning. Among the Mg^2+^-wasting-related disorders are osteoporosis, abnormal brain development, neurological alterations, immunodeficiency, impaired motility of sperm, diabetes, hypertension, cancer, and several rare diseases such as familial hypomagnesemia or Jalili Syndrome [6,9,12,13,14,15,16,17,18,19].

Among the most intriguing magnesiotropic proteins are the cyclin and CBS domain divalent metal cation transport mediators (CNNMs). The CNNMs, formerly known as “ancient conserved domain proteins” (ACDPs), were discovered in 2003 [20], and were categorized in accordance with the presence of an ancient motif and a weak sequence similarity with cyclins, although no cyclin-related function has been proven so far. As now specified in their revisited nomenclature, CNNMs also contain a cystathionine β-synthase (CBS) domain pair [21], which, upon binding to different molecular partners, is thought to regulate their overall activity. In humans, the cyclin M (CNNM) family encompasses four members (CNNM1–4), whose exact function is currently being investigated. Some authors claim CNNMs to be direct transporters that extrude Mg^2+^ ions from the cell by exchanging it with Na^+^ ions [18], whereas others think that their function may not involve direct membrane transport, acting either as intracellular Mg^2+^ sensors or as Mg^2+^ homeostatic mediators of other not-yet-identified transcellular transporters [22,23,24,25,26]. The organ distribution and expression pattern of the CNNMs are divergent (Table 1) [22,26,27]. CNNM1 appears to act as a cytosolic copper chaperone [28] and is mainly found in the testis and brain [11,20,22,29,30], whereas low expression levels are found in the stomach, kidney, skeletal muscles, heart, lungs, liver, small intestine, colon, and spleen [29]. In the testis, its expression is limited to c-KIT- and OCT3/4-positive cells, which are spermatogonial stem cells (SSCs) or early spermatogonial cells [31], thus suggesting a role as a regulator of germ cell division and differentiation [29]. CNNM2, the most studied member and whose actual function is heavily debated either as the long sought basolateral Mg^2+^ extruder at the renal distal convoluted tubule (DCT) [32,33], or alternatively as a Mg^2+^ homeostatic factor [25], is ubiquitous, although mostly expressed in the brain, kidney, liver, and heart [20,27,34,35]. Lower expression levels are also detected in odontoblasts, the small intestine, and colon [34,36] (Table 1). Some studies have reported lower amounts of CNNM2 in the thick ascending limb of Henle’s loop (TAL) and in the connecting tubule (CNT) [22], suggesting its potential role in paracellular Mg^2+^ reabsorption [37]. CNNM3 is ubiquitous, but mostly present in the lung, spleen, and heart [20,27], and is barely detected in skeletal muscles [20] or odontoblasts [36]. CNNM4, postulated to mediate the basolateral extrusion of Mg^2+^ through the exchange of Na^+^ [18,23], is highly abundant in the intestinal tract [18,23,27], in mature ameloblasts [23], odontoblasts [36], and in sperm [19] (Table 1).

In agreement with the ubiquitous but differential distribution of the four CNNM members, their impairment is linked to the development of distinct pathologies related to magnesium wasting in different organs (Table 1). For example, mutations in CNNM2 have been shown to cause familial primary hypomagnesemia with normocalciuria and normocalcemia [22], a recessively inherited disease whose clinical symptoms are weakness of the limbs, vertigo, headaches, seizures, brisk tendon reflexes, and mild to moderate psychomotor delay. The impairment of CNNM2 activity has been linked to mental retardation and neuro-psychiatric disorders (e.g., schizophrenia) [38] as well as with brain development anomalies [17]. In this regard, a CNNM2 deletion is embryonically lethal in mice [39], and heterozygous Cnnm2^+/−^ mice show lower blood pressure. Additional studies have related CNNM2 to diabetes [40], obesity [41], and infertility [42] (Table 1).

**Figure 1:**
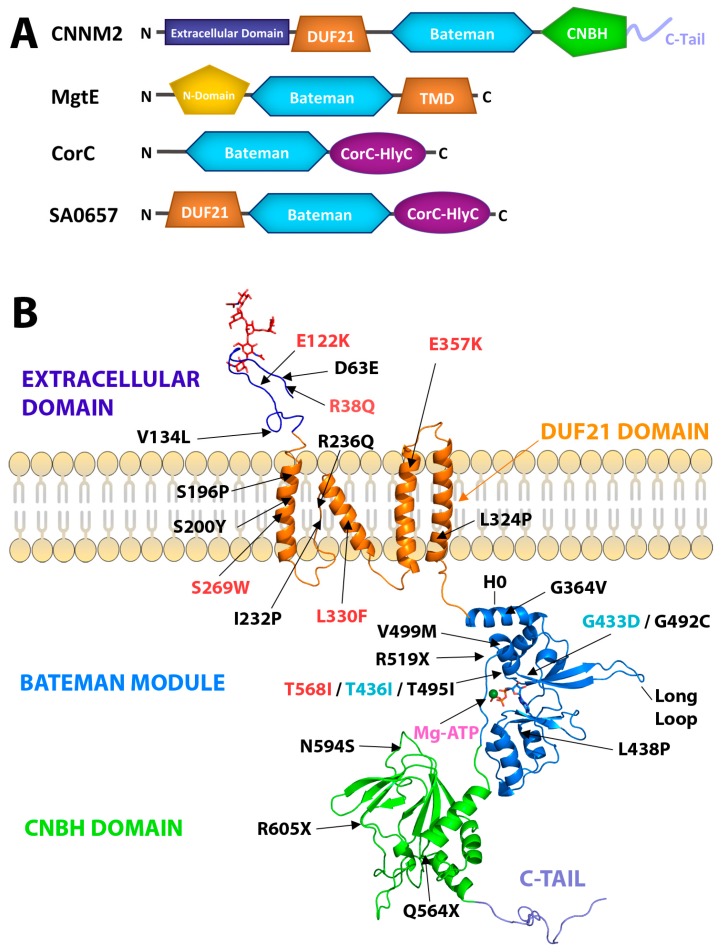
Structure of CNNMs. (**A**) Domain distribution in a related cystathionine β-synthase CBS domain containing metal ion transporters, including CNNM2. The Bateman module (in blue) plays a regulatory role in all the proteins shown. The DUF21 domain represents a major part of the transmembrane domain (TMD) (orange) and is thought to contain from three to four α-helices. CNBH domain is in green. The N-terminal extracellular region of CNNM2 is predicted to be β-strand-enriched (deep purple). The bacterial MgtE channel shares the presence of a CorC-HlyC domain (purple) with CorC and with other proteins such as the bacterial SA0657 protein [57]. (**B**) The panel shows in ribbons the three-dimensional structure of the isolated domains. The structures of the Bateman module and the CNBH domain were extracted from crystallographic data [43,45,58,59]. The representation of the DUF21 domain corresponds to an in silico model [22]. The CNNM representation is as follows: The N-terminal extracellular domain (dark blue) shows the glycosylation in red; the transmembrane α-helixes are represented in orange. In the intracellular region, the Bateman module (PDB code: 4P1O) is represented in blue; bound MgATP is in pink. The CNBH domain (PDB code: 6DJ3) is in green. The unstructured terminal C-tail is in purple. The locations of all known pathological mutations reported for CNNMs are indicated by arrows and in different colors depending on the variant protein: CNNM2 (red), CNNM3 (cyan), and CNNM4 (black). Helix H0 connects the Bateman module with the DUF21 domain.

Mutations in CNNM4 are the cause of Jalili syndrome (JS), another inherited disease characterized by the association of amelogenesis imperfecta (AI) and cone-rod dystrophy (CRD) [16,47]. CNNM4 in patients with JS was first identified in 1988, and since then, 17 different mutations have been reported in patients with this syndrome (Figure 1, Table 2). Although the pathogenic mutations identified in CNNMs are distributed along their entire amino acid chain, a significant number of these mutations concentrate in the DUF21 (domain of unknown function-21) and CBS domains (Figure 1, Table 2) [48]. The recently identified association between the CNNM family and the highly oncogenic phosphatases of the regenerating liver (PRLs) to form stable complexes that promote tumor progression and metastasis, have set the focus on CNNMs as new attractive therapeutic targets for cancer treatment [18,24,43,44,45,46].

The objective of this review was not to outline the specific functions of the different CNNMs, since additional studies are still necessary for objective assessments, but to summarize the current knowledge on the overall architecture of these complex proteins, highlighting their similarities and differences with respect to their closely related homologs. This work also summarizes the known ligands and interacting molecular partners, as well as the structural changes induced in the CNNMs by these molecules. These data may help the general reader evaluate the potential capacity (or incapacity) of CNNMs to transport Mg^2+^ ions through the cell membranes and develop future strategies to modulate their activity in a specific manner in different organs, thus contributing to alleviating a wide variety of pathologies.

## 2. Structure of CNNMs

**Figure 2:**
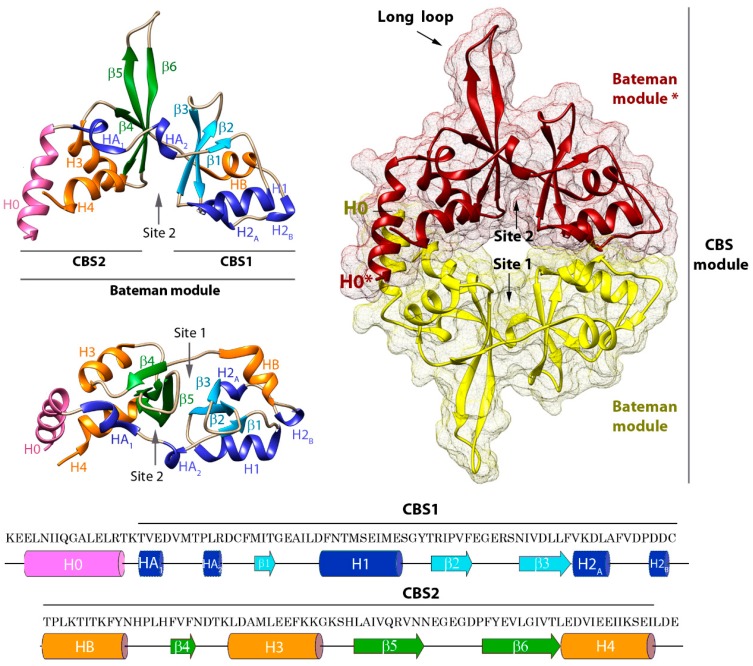
Structure of the Bateman domain of CNNM2. (**left**) Two views of the structural elements configuring the Bateman module of CNNM2 (in ribbons). The main symmetric cavities S1 and S2 are indicated. S2 hosts MgATP (**right**) CBS module: parallel (head-to-head) dimeric association of Bateman modules. The S2 cavities from the complementary subunits are located on opposite sides of the disc. (**bottom**) Amino acid sequence of human CNNM2. The secondary elements are indicated.

Structurally, CNNMs are complex modular proteins that contain four independent domains connected by linkers of different length (Figure 1). The N-terminal region is extracellular and shares the lowest amino acid sequence similarity (~19%) among the four members encoded in the human genome. This region contains one transmembrane α-helix followed by a predicted β-stranded enriched zone. It also includes a long signal peptide (64 amino acids in length in the case of CNNM2) that allows cleavage of the N-terminal section by the signal peptidase complex (SPC, a protein complex expressed on the endoplasmic reticulum (ER) membrane known to be involved in signal peptide cleavage) [27], and a conserved asparagine residue (N112, N73, and N85 in CNNM2, CNNM3, and CNNM4, respectively) that provides a glycosylation site necessary for the proper plasma membrane location in at least three members of the CNNM family [27]. Following the first extracellular module is a DUF21 membrane spanning domain (Pfam code PF01595), of which almost nothing is known apart from its amino acid composition and its distribution across the sequenced genomes. DUF21 domains are detected in plant proteins fused to CBS domains [60] and in the yeast CNNM homolog, MAM3p (Figure 1) [61], but their structure and properties remain undetermined. In human CNNMs, the DUF21 domain is predicted to contain four transmembrane α-helices, of which one is very short. The amino acid sequence similarity in this region increases to 45%, being higher between CNNM2 and CNNM4 (~79%), and lower between the latter and CNNM3 (~30%). Whether these differences confer particular ion-filtering capabilities and specific mechanisms of transport to each CNNM member remain to be determined experimentally. The polypeptide segment that follows represents the large cytosolic region of CNNMs and includes two independent domains that do not (or barely) interact with each other (Figure 1) [45]. These two domains are linked by a long unstructured peptide that shares moderate sequence identity (~40%). The first domain, known as the ‘Bateman module’, consists of two consecutive cystathionine β-synthase (CBS) motifs (Figure 2) [21,62], and represents the most conserved region of the entire protein (~78% on average and 90% between CNNM2 and CNNM4). A long α-helix (H0) preceding the CBS1 motif links the Bateman module with the DUF21 domain, allowing a direct communication between the cytosolic and the transmembrane regions. The overall three-dimensional appearance of the Bateman module is similar to a bean seed (Figure 2). Each lobe is occupied by a CBS motif, which in turn is formed by a three-stranded β-sheet and two α-helices packed according to the sequence β1-α1-β2-β3-α2 along with a flexible linker that precedes the first β-strand [21,43,44,45,58,62,63]. The central part of the Bateman module shows two main symmetrically oriented cavities (named as S1 and S2) whose side and back walls are formed by the β-sheets of the CBS motifs (Figure 2). As described below, the unique combination of amino acids decorating the walls of these clefts in the CNNMs allows S2 but disables S1 to host adenosine derivatives inside [21,58,62].

**Figure 3:**
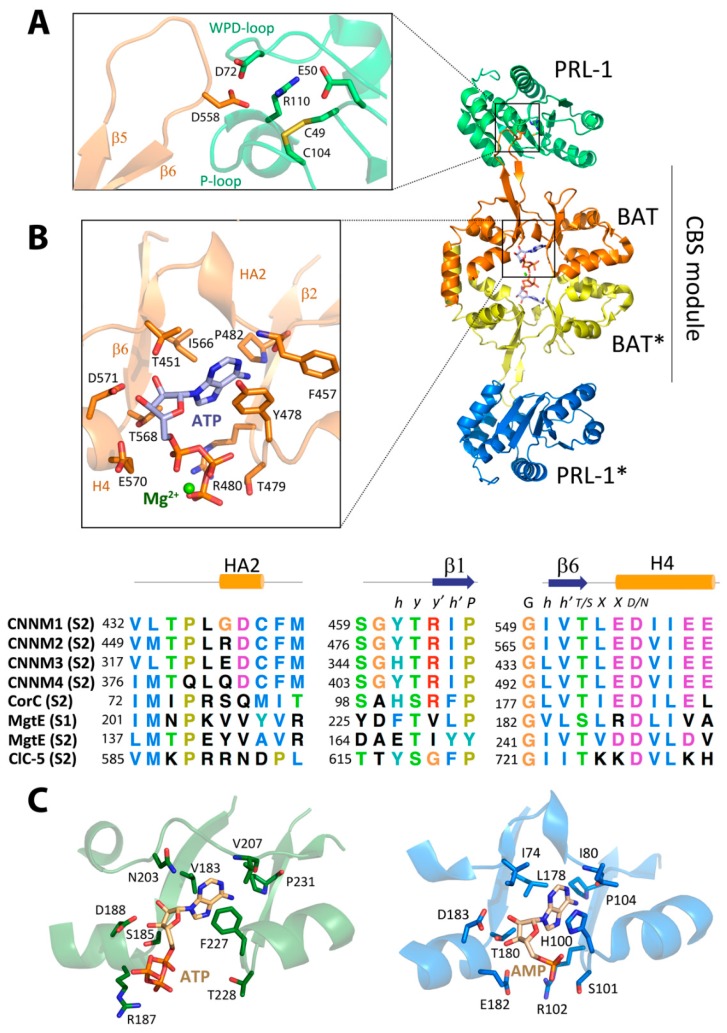
Structure of the CNNM-PRL complex. The picture shows the crystal structure of the CBS module of CNNM2 (PDB code: 5LXQ) (the two Bateman modules are in yellow and orange) in complex with PRL-1 (in green and blue). As shown, the CBS module adopts a flat conformation. MgATP (in sticks) occupies the complementary S2 cavities. (**A**) Detail of the main interactions between CNNM2 and PRL-1. D558 from CNNM2 (orange) enters the catalytic cavity of PRL-1 (green); (**B**) MgATP-binding site (cavity S2) in CNNM2. The main residues involved in the interaction with ATP are represented in sticks. (**Bottom**) Sequence alignment of the three main blocks forming the walls of the S2 site. The conserved motif *h y y′ h′ P* (where *h* is hydrophobic, *y* is whatever, and *P* is proline) stabilizes the adenine ring and favors the presence of adenine-derived nucleotides. Conserved T568 and D571 interact with the hydroxyl group of the ribose ring and belong to the conserved motif *G h h′ T/S X X D/N* (where *h* is hydrophobic, and *X* is any residue). The electrostatic repulsion exerted by residues E570, D571, and E572 is partially neutralized by Mg^2+^. Nucleotides bind to Site 2 (S2) in CNNMs, CorC, and ClCs, whereas MgtE binds ATP in Site 1 (S1). However, as shown in the alignment, conserved motifs are also present in S1 of MgTE. (**C**) Nucleotide-binding sites in MgtE and CorC (adapted from Tomita et al., 2017). (**left**) Site S1 of MgtE (represented in green), ATP is represented by sticks (PDB code: 5X9G). The conserved threonine of the *G h h′ T/S X X D/N* motif is substituted by a serine, and the conserved glutamate (E570 in CNNM2) is substituted by an arginine that interacts with the triphosphate chain of the ATP. The hydrophobic environment that stabilizes the position of the adenine ring is also present (motif *h y y′ h′ P*). (**right**) Site S2 of CorC (blue) bound to AMP (PDB code: 5YZ2) [69].

In agreement with the most common behavior of CBS domain proteins [21,62], the Bateman module of CNNMs associates with itself, forming a disc-like symmetric dimer that was first named by Mahmood as the CBS module (Figure 2) [64]. Following the most widespread tendency, the interacting Bateman modules are oriented parallel, with their N-terminal ends pointing in the same direction toward the transmembranous zone. Besides supporting the dimer being the most probable biological unit in CNNMs, this sort of arrangement has several consequences. First, this arrangement causes the complementary CBS2 motifs (and thus, the H0 helices that connect them to the DUF21 domains) to face each other, close to the cytoplasmic side of the cell membrane, while keeping the CBS1 motifs away from it (Figure 2). Secondly, the complementary S2 cavities from the two subunits lie on opposite sides of the disc, symmetrically balancing what happens on both sides of the dimer when ligands bind the protein. Third, it ensures a symmetric and coordinated communication of the complementary subunits with the region spanning the cell membrane (DUF21 domain) and with the rest of the domains that configure the entire transporter. Finally, it helps to select the identity of the ligands to be potentially hosted at the cavities, their orientation inside, and the strength of their interaction [58]. A unique feature that differentiates the Bateman module of CNNMs from that found in other proteins is the presence of an extended loop between the last two β-strands of the CBS2 motif (Figure 1 and Figure 2). This anomalous long loop undermines the internal two-fold symmetry existing between the two CBS motifs of the Bateman module, and confers special prominence to CBS2 to interact with other molecular partners (Figure 3) [43,44,45]. Phylogenetic studies revealed that the presence of this elongated segment is a common feature of all mammalian CNNMs, but is also observed in homologs from less evolved organisms. Its presence accompanies the coding of a PRL counterpart in the above 150 genomes analyzed, suggesting a co-evolution of the two proteins in all life kingdoms [24]. Again, the parallel orientation of the CBS module ensures that partner proteins interacting with CNNMs through the long loop remain equidistant from the transmembrane zone. This is key for interacting molecules, such as PRLs (Figure 3) (described below) [43,44,45], which contain a prenylation site that helps maintain their attachment to the cell membrane [65].

**Figure 4:**
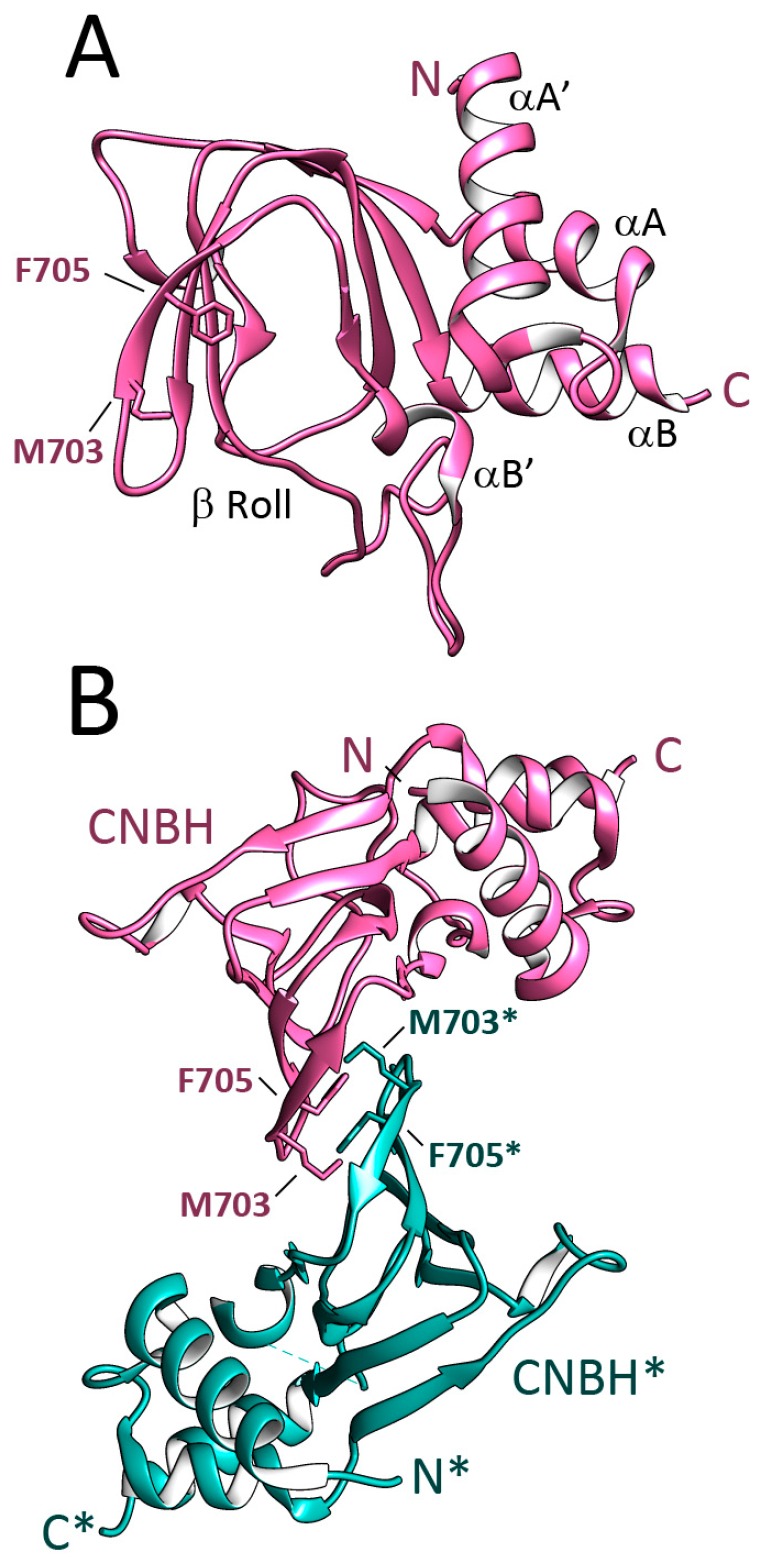
Crystal Structure of the CNBH of CNNM2. (Figure prepared using atom coordinates from PDB code 6DJ3 [59]. (**A**) The structure shows a central eight-stranded β roll preceded by two α-helixes in the N-terminal part (αA′ and αA), and is followed by a C-terminal α helix called αB. The small αB helix is inserted in the beta roll. Residue F705 and some other bulky residues autoinhibit the domain and impair nucleotide binding [59]; (**B**) The CNBH domain associates in dimers. The complementary subunits are depicted in pink and green. Residues F705 and M703 are relevant in the subunits association.

The C-terminal end of the CNNMs includes a cyclic nucleotide monophosphate (cNMP) binding-like domain (Pfam code PF00027), which shares an amino acid identity of 5% with the CNNMs. This module is followed by a long unstructured C-tail and was recently renamed CNBH (see below) (Figure 1). The removal of this zone completely blocks the magnesium extrusion capacity of CNNM4 [59]. The overall fold of the CNBH module of CNNM2 and CNNM3 was recently elucidated by Gehring et al. at 2.6 and 1.9 Å resolution, respectively [59], using engineered protein constructs with remodeled flexible zones, including a long loop located in the β-roll, and the elimination of the following C-terminal tail (Figure 4). The crystal structures revealed that the formerly-called cNMP domain is in fact a cyclic nucleotide monophosphate-binding homology (CNBH) domain, thus a structural domain that shows a similar fold to that of cyclic nucleotide binding domains (CNBDs), but without the binding capacity for cyclic nucleotides (like cAMP or cGMP) of the latter. The CNBH domain of CNNMs is formed by two N-terminal α-helices (αA′, αA), followed by an eight-stranded β-roll and a C-terminal α-helix (αB) (Figure 4). Several features differentiate the CNBH module of CNNMs from the classic cyclic nucleotide-binding domains (CNBDs) or the CNBD-homology domains (CNBDHs or CNBHs) found in other proteins. The first difference is the central β-roll, which presents a pocket in classical CNBDs suitable to host cAMP or cGMP. In the CNNMs, this location is sterically hindered by bulky residues that invade the location of any potential ligand [59]. The inability of the CNNMs to bind cyclic nucleotides was confirmed using thermal shift assays (TSA) and nuclear magnetic resonance (NMR) as a more sensitive technique [59]. A second difference is the loop that connects strand β7 with helix αB, which appears significantly longer in CNNMs (30–70 residues) than in classical CNBDs or CNBDHs. Finally, the C-terminal tail is unstructured in the CNNMs, whereas it remains well folded in other proteins. In the CNBDs, the C-tail usually includes a long α-helix (commonly known as αC) that contains a conserved arginine residue that stabilizes the cyclic nucleotide inside the β-roll cavity [66,67]. In contrast, the classical CNBDHs have a short β-strand following helix αC that contains a Tyr-Asn-Leu motif that enters the potential nucleotide site acting as an intrinsic autoinhibiting ligand [68]. Gehring et al. found that the CNBHs from CNNM2 and CNNM3 exist as homodimers that are detectable both, in the crystals (Figure 4), and in solution. In agreement with the formerly observed dimerization of the Bateman module of CNNMs, these findings support this oligomer as the most probable functional unit of the full-length protein. The analysis of the neighboring molecules in the crystals revealed that the inter-subunits interface is stabilized by hydrophobic contacts between residues located at the loop connecting strands β4–β5 of the β-roll (Figure 4). Substitution of these residues completely shatters the dimer, highlighting their key role in the association of complementary CNBH domains. Several observations underline the need for further experiments to unequivocally identify the actual functional unit of these protein modules. Among these observations are (1) the presence of monomeric entities in solution, in ratios that vary with the protein concentration [59], and (2) the fact that mutants in which the dimerization of the CNBH dimer is impaired are even more active than the wt-protein.

## 3. CNNM Ligands and Interacting Partners

The identification of CNNM-interacting molecules has barely been explored. The partners identified so far can basically be classified as: (1) molecules that intervene in the cellular location of the CNNMs and (2) molecules that modulate their transport activity. The latter can be further subdivided into two categories: (1) small ligands (i.e., nucleotides, metal ions) and (2) large macromolecules (proteins) (Table 3).

### 3.1. Modulators of the CNNMs’ Cellular Location 

Previous findings demonstrating the basolateral localization of CNNMs in the renal [22] and intestinal epithelia [23] prompted Hirata et al. to examine the potential role of clathrin adaptor proteins (AP) in the localization of CNNM4 [42], as APs are related to cargo recognition through the direct binding of signal motifs in the corresponding targets [70,71,72]. The AP-1 proteins contain four subunits: γ, β1, σ1, and μ1. Of them, μ1A and μ1B were found to complement each other in recognizing CNNM4 and direct its basolateral sorting by recognizing three independent dileucine motifs (L575/576, L758/759, and L765/766), located at the C-terminal CNBH domain of the CNNMs [42]. These findings assign, for the first time, a concrete role of the CNBH domain in the CNNMs function.

### 3.2. Small Molecules That Modulate CNNMs’ Activity

The cystathionine β-synthase (CBS) motifs are characterized by their capacity to host adenosine derivatives that, upon binding, trigger conformational changes in the proteins in which they are inserted to modulate their overall activity [21,62,63,73]. Similarly, the cyclic nucleotide monophosphate-binding domains (CNBDs) contained in ion channels and cNMP-dependent kinases are known to be subject to structural modifications induced by the interaction with second messengers like cAMP or cGMP [74]. With these antecedents, the identification of a Bateman module and a CNBH domain in the cytoplasmic region of CNNMs (Figure 1) immediately suggested their potential regulation by small molecules [27]. The first experimental evidence was provided by Hirata et al. [42], who used Surface Plasmon Resonance (SPR) techniques to detect binding of ATP to the Bateman module of CNNM2 (*K*d ≈ 160 μM) dependent on the presence of Mg^2+^. Patient mutation T568I, causing familial hypomagnesemia [22], was found to abolish this interaction, confirming the idea that ATP regulates the activity of the transporter [42]. Almost in parallel, and using a more sensitive NMR approach, Corral-Rodríguez et al. confirmed the Mg^2+^-dependent interaction of CNNM2 with ATP. In contrast with the Hirata et al. findings, interaction with AMP and ADP was also evidenced [58]. The study by Corral-Rodríguez et al. revealed a weak affinity of CNNM2 for ATP (*K*d > 10^−2^ M), even in the presence of Mg^2+^. The spectral protein signals affected by ATP differed from those affected in the presence of Mg^2+^ alone, indicating different sites of interaction [58].

### 3.3. ATP Binding Site of CNNMs Differs from That of Its Homologs

Further X-ray crystallographic studies led to the identification of cavity S2 as the unique ATP site in CNNM2 [45,58], and revealed unique characteristics that distinguish it from the nucleotide-binding site of related CBS domain-containing proteins such as MgtE (which binds ATP at S1) [75], CorC (AMP at S2) [69], or CLC-5 (ATP at S2) (Figure 3) [76]. These particular features not only determine different affinities for the adenosine derivatives, but also outline the specific changes triggered by each concrete nucleotide in the target proteins. In all cases, the canonical cavities of their Bateman modules are composed of three different structural blocks: (1) the flexible linker containing the short α-helix HA2 that precedes strand β1 of the CBS1 motif, (2) strand β2 of CBS1, and (3) strand β6 of CBS2 together with the first two turns of the following α-helix (H4) (Figure 2 and Figure 3) [45,58]. The first two blocks provide a hydrophobic pocket that accommodates and orients the adenine ring and fixes the orientation of the ribose ring within the cavity. However, these elements are insufficient to discriminate among different adenosyl derivatives within the cavity (Figure 3). Among the residues involved are a conserved phenylalanine located at the loop that precedes strand β1 (F440, F457, F325, and F384 in CNNM1 to 4, respectively) and a tyrosine that precedes strand β2 (Y461, Y478, and Y405 in CNNM1, 2, and 4, respectively) (Figure 3). In CNNM3, residue H346 occupies the equivalent position of the conserved tyrosine (Figure 3). The orientation of the ribose ring of ATP is fixed by three main residues: the first is a conserved threonine belonging to the first structural block that precedes the short α-helix HA2 (T434, T451, T319, and T378 in CNNM1 to 4, respectively) [45,58]. The second is a threonine (T568 in CNNM2; for equivalents, see Figure 3) located at the end of strand β6 in the third block. The third residue is a conserved aspartate (D571 in CNNM2) located at the first turn of the last α-helix (H4) of the CBS2 motif (third structural block) (Figure 3). These three amino acids form an extensive H-bond network with the hydroxyls of ribose that, together with the formerly mentioned hydrophobic pocket, orient the adenosyl moiety within the cavity. The third structural block configuring the site marks the difference in terms of nucleotide selection and binding affinity across the different homolog proteins. For example, in both the CNNMs and CorC, this block includes a rarely observed acidic cluster at the first two turns of helix H4 (Figure 3). This cluster, represented by residues E570, D571, and E574 in CNNM2 (the equivalents for other CNNMs and CorC can be found in Figure 3), exerts an electrostatic repulsion that disfavors the interaction with negatively charged groups (i.e., the polyphosphate chain of ATP). Reduction in the negative charge in the ligand (i.e., a lower number of phosphate groups), or alternatively the presence of divalent cations bound to the nucleotide (MgATP), neutralize this repulsion, thus increasing the affinity of the nucleotide for the protein. This reduction not only explains the observed Mg^2+^-dependent interaction of ATP with CNNMs, but also the higher affinity of AMP than ATP for CNNM2 [58]. The reduced electrostatic repulsion might also justify why just the AMP-bound form of CorC (PDB code 4HG0), and not the corresponding ATP complex, has been reported so far (Figure 3). In contrast with the CNNMs and CorC, an arginine or a lysine at the position of residue E570 in CNNM2 stabilizes the polyphosphate chain of ATP in MgtE and ClC-5, respectively, thus favoring the interaction in these proteins (Figure 3).

### 3.4. Large Modulators of CNNMs’ Activity

In 2014, two independent studies by Tremblay et al. [24] and Miki et al. [18] revolutionized the field after reporting that a major mechanism of oncogenesis is mediated by the interaction of CNNMs with the phosphatases of the regenerating liver (PRLs). PRLs are considered the most oncogenic phosphotyrosine phosphatases (PTPs) subfamily and they are highly over-expressed in solid tumors and haematological cancers [77,78,79]. Both research groups found that the formation of PRL-CNNM complexes leads to a significant increase in the intracellular Mg^2+^ levels that are used by the tumor to proliferate and migrate [23,24]. The basis of such interaction was firstly postulated by Tremblay and colleagues [24,46], and confirmed experimentally soon after by Gimenez-Mascarell et al. who used X-ray crystallography prove that a highly conserved aspartate residue (D558 in CNNM2), located at the tip of the extended loop of the CBS2 domain of CNNM2, invades (and inhibits) the catalytic cavity of PRL-1, acting as a pseudosubstrate that is crucial for the CNNM∙PRL interaction (Figure 3) [45]. Substitution of the conserved aspartate blocks the complex formation and results in an antiproliferative effect on human breast cancer cells [46]. Gehring et al. later confirmed these results in other CNNM and PRL members [43,44]. It still remains unclear whether the oncogenic effect caused upon formation of the complex is due to the CNNM-induced inhibition of the phosphatase, to the PRL-induced inhibition of the CNNM transporter, or perhaps, to both. The following section reviews the conformational changes occurring in the two proteins when the complex is formed.

## 4. Ligand-Induced Conformational Changes

**Figure 5:**
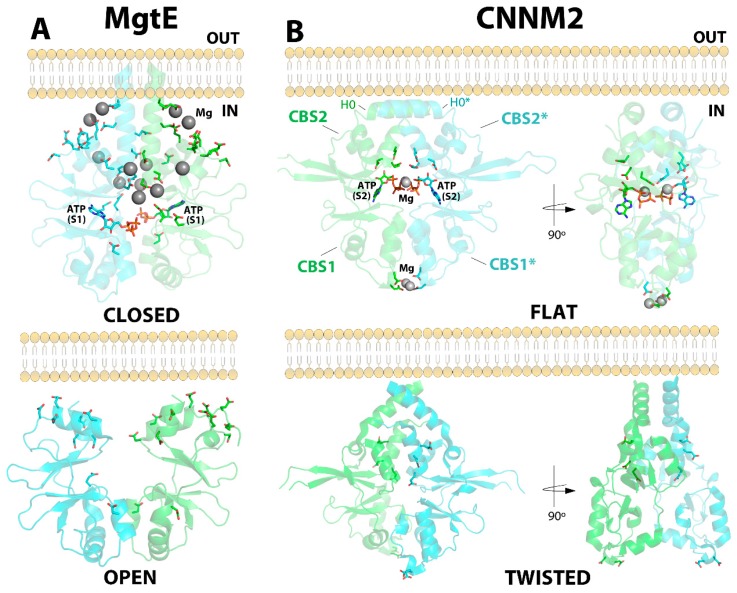
Structural comparison of CNNM2 and MgtE. (**A**) Structure of the CBS module of MgtE in its two different conformations: (**top**) closed and (**bottom**) open. Mg^2+^ ions are represented by grey spheres. The acidic clusters are represented by sticks. Upon binding of Mg^2+^ ions, the CBS module evolves from its open state (bottom) to its closed Mg^2+^-bound conformation (top). (**B**) CNNM2 may adopt two different conformations: Flat (top right) when bound to MgATP, and twisted (bottom right) in the absence of a nucleotide. The main residues involved in ATP and Mg^2+^ binding are represented by sticks.

The transmembranous domain and the intrinsic flexibility of the extended loops and connecting linkers present in all CNNMs have traditionally converted their crystallization through a difficult task that restricts our current structural knowledge to the more compact regions of the isolated intracellular modules. The scarce data indicate that CNNMs undergo conformational changes upon binding their different ligands. To elucidate the mechanisms through which these structural changes mediate the transport of magnesium through the cell membranes, it is necessary to know the structure of both the apo- (unbound) conformer and the different holo-complexes with the interacting partners. This complicated challenge has only been overcome successfully for the Bateman module, which represents the most widely studied region of CNNM proteins [42,43,44,45,58,59]. As mentioned above, the Bateman module of CNNMs binds ATP in a Mg^2+^-dependent manner in the S2 cavity. In the absence of a bound nucleotide, the two CBS motifs of the Bateman module maintain a relative orientation that is determined through a mixed network of hydrophobic, H-bond, and salt link interactions between the residues that form the walls of the two canonical cavities, S1 and S2. Among these interactions, the salt bridge formed by an arginine at position 480 and a glutamate at position 570 in CNNM2 is highlighted, as well as a network of H-bonds centered on threonine at position 568 (Figure 3). These residues are conserved in all CNNMs and in the CorC protein (Figure 3), and in the CNNMs cause a contortion of the Bateman module, which results in a “twisted” conformation of the CBS module [58]. Upon binding at site S2, MgATP disrupts all these interactions and establishes new ones with residues from both CBS motifs at opposite walls of the cavity. These sequences of events not only release the former ties that maintained the original orientation between the two CBS motifs, but trigger the shift of several secondary elements, among which is the H0 helix connecting the Bateman module with the transmembrane domain (Figure 1). Of note, the location of the S2 cavities on opposite sides of the CBS module permits binding of ATP at the two complementary Bateman modules in the dimer, thus making the process structurally symmetric. The consequence of these processes is the flattening of the CBS module, that adopts a disc-like flat conformation (Figure 5, Supplemental movies 1 and 2) [45,58]. In an apparent contradiction, the mutation of residue T568I, found in the CNNM2 protein of patients suffering familial hypomagnesemia, mimics the effect of the nucleotide in the Bateman module, despite this amino acid substitution impairing ATP binding [58]. The flattening of the disc in the protein mutant is conformationally irreversible, as the disruption of the H-bond network centered on residue T568 is permanent [58].

A similar flattening of the CBS module was reported by Gimenez-Mascarell et al. after solving the crystal structure of CNNM2 in complex with PRL-1. In this case, the change in the relative orientation of the CBS motifs appears to be triggered by the electrostatic attraction between the surfaces of the two interacting proteins [45]. The authors postulated that the CBS motifs shift, after the CNNM anchors, to the catalytic cavity of the target PRL through the conserved aspartate located at the extended loop of the CBS2 motif. In summary, binding of either MgATP or PRL proteins to the Bateman module of CNNMs triggers a rotation of the CBS motifs that flattens the CBS module. If sustained over time, this flat conformation is thought to deactivate the extrusion capacity of the transporter [45]. In agreement with this hypothesis, the CNNM2 T568I mutation (known to permanently lock the CBS module in the flat conformation) [58] impairs the basolateral extrusion of Mg^2+^ at the renal DCT, thus causing hypomagnesemia.

An open-to-close conformational change is triggered upon progressive binding of Mg^2+^ atoms to the CBS module of the bacterial channel MgtE [75,80,81]. In the absence of Mg^2+^ ions, the interfacial helices from complementary CBS2 motifs (the functional unit of MgtE is a dimer) remain distant from each other due to the electrostatic repulsion exerted by acidic residues located at their interfacial α-helices (represented by sticks in Figure 5). This conformation keeps the channel open and allows access of Mg^2+^ ions through the cell membrane. Progressive binding of Mg^2+^ to the acidic clusters (shown in sticks in Figure 5) decreases such electrostatic repulsion and permits an approximation of the CBS2 motifs, which produces the formation of a flat CBS module, similar to that described for CNNM2. Several studies have proved that this flat arrangement closes the ion-conducting pore, thus locking the closed state of the channel and interrupting Mg^2+^ uptake (Figure 5). The apo-conformation of the CBS module of MgtE is structurally different from the twisted disc observed in the CNNMs, but the Mg^2+^-bound form clearly reproduces the overall pattern observed in the CNNMs [80,81]. Of note, recent findings by Tomita et al. proved that ATP binding to the Bateman module enhances the intracellular domain affinity for Mg^2+^ within physiological concentrations, thus enabling MgtE to function as an in vivo Mg^2+^ sensor [75]. 

## 5. Conclusions

This review summarized the current three-dimensional knowledge about the CNNM family of magnesium homeostatic factors. Their exact role remains unclear [22,23,25,82], and the sparse information is insufficient to clarify which of the different (and in some cases, contradictory) functional models proposed better fits its actual function. Several structural characteristics that support its direct involvement in the Mg^2+^ transport process have recently been revealed. Among them are (1) the presence of a transmembrane domain potentially capable of passing metal ions through the cell membranes; (2) the presence of a regulatory Bateman domain directly connected to the TMD in a similar fashion to other transport proteins such as MgtE, CorC, or the CLC family of chloride channels; (3) the ATP- and Mg^2+^-binding capacity of the Bateman module, and the conformational changes associated to the interaction with these ligands, formerly observed in other channels; (4) the presence of a C-terminal CNBH domain, whose removal impairs Mg^2+^ extrusion; (5) the structural role exerted by hypomagnesemia causing mutations, such as CNNM2 T568I, which impair Mg^2+^ extrusion at the DCT; and (6) the lack of identified interacting transporters that could potentially be regulated by CNNMS, thus positioning the latter as simple modulators of their activity. Evidence not supporting the activity of CNNMs as direct transporters includes (1) the scarce number of Mg^2+^ binding sites located in the regulatory Bateman module, (2) the moderate number of transmembrane α-helices in the DUF21 domain, and (3) the low oligomerization degree (a dimer) in comparison with other channels and transporters.

The behavior of the MgtE channel and CNNM2 are opposite in terms of ATP- and Mg^2+^-binding mode. MgtE binds Mg^2+^ in an ATP-dependent manner, whereas CNNM2 only interacts with ATP at a certain (mM) Mg^2+^ concentration. These striking features make it tempting to speculate that these opposing affinities for the two ligands could be related to the inverse abilities of these homeostatic factors to transport magnesium ions in opposite directions across cell membranes, thus supporting the direct role of CNNMs in Mg^2+^ extrusion. 

The recent structural advances have contributed to our early understanding of the mechanisms underlying the CNNMs function, as well as their interaction with different molecules. Answering the still significant number of unanswered questions will help our ability to modulate the activity of these relevant machineries and use them as promising targets to treat Mg-related disorders and several types of cancer.

## Figures and Tables

**Table 1:** Tissue distribution of the CNNM family. High expression levels are marked in bold.

Protein	Localization	References
CNNM1	stomach, kidney, skeletal muscles, heart, lungs, liver, colon, spleen, small intestine, **brain, testis**	[11,20,24,29,43,44]
CNNM2	odontoblasts, small intestine, colon, **kidney, lung, spleen, testis, brain, liver, heart**	[20,24,27,34,35,36,43,44,45]
CNNM3	odontoblasts, skeletal muscles, **kidney, brain, lung, spleen, heart, liver**	[20,24,27,36,43,44,46]
CNNM4	**odontoblasts, colon, mature ameloblasts, sperm** **gastrointestinal tract**	[18,23,24,27,36,43,44]

**Table 2:** Pathological mutations found in CNNMs identified in patients. Asterisk indicates a premature stop codon. “X” indicates the amino acid after which the protein is truncated. CNBH refers to cyclic-nucleotide-binding homology domain.

Protein	Mutation	Domain	Pathology	References
**CNNM2**	R38Q, E122K	Extracellular	Hypomagnesemia	[17,22]
	S269W, L330F, E357K	DUF21	Hypomagnesemia	[17]
	T568I	Bateman module	Hypomagnesemia	[46]
**CNNM4**	D63E	Extracellular	Jalili Syndrome	[49]
	S196P, S200Y, I232P, R236Q, L324P	DUF21	Jalili Syndrome	[14,47,50]
	G364V, G492C, L438P, T495I, V499M	Bateman Module	Jalili Syndrome	[14,50,51,52,53]
	R519X	Linker Bateman-CNBH	Jalili Syndrome	[54]
	N594S, Q564X, R605X, T581 *	CNBH	Jalili Syndrome	[47,55,56]

**Table 3:** Available structural data on the CNNM family.

Protein	Domain	Partner	PDB Code	Reference
CNNM2	Bateman module	-/AMP/ADP/ATP/Mg^2+^	4IYS,4P1O, 4P1G, 4IY0, 4IY4	[58]
	Bateman module	PRL-1/ATP, Zn^2+^	5LXQ, 5MMZ	[45]
	CNBH	--	6DJ3	[59]
CNNM3	Bateman module	PRL-2	5K22, 5K23, 5K24, 5K25	[43,44]
	Bateman module	PRL-3	5TSR	[44]
	CNBH	--	6DFD	[59]
CNNM4	Bateman module	--	4IY3	[58]

## Supplementary Materials

Supplementary materials can be found at https://www.mdpi.com/1422-0067/20/5/1135/s1. Supplemental Movies S1 and S2: The movies show two different views of the CBS module and the conformational change induced by MgATP upon binding to the S2 cavity of the complementary Bateman modules (in red and yellow, respectively).

## References

[1] Maguire M.E., Cowan J.A. (2002). Magnesium chemistry and biochemistry. Biometals.

[2] Gwanyanya A., Amuzescu B., Zakharov S.I., Macianskiene R., Sipido K.R., Bolotina V.M., Vereecke J., Mubagwa K. (2004). Magnesium-inhibited, TRPM6/7-like channel in cardiac myocytes: Permeation of divalent cations and pH-mediated regulation. J. Physiol..

[3] Saris N.E., Mervaala E., Karppanen H., Khawaja J.A., Lewenstam A. (2000). Magnesium. An update on physiological, clinical and analytical aspects. Clin. Chim. Acta.

[4] Volpe S.L. (2013). Magnesium in disease prevention and overall health. Adv. Nutr..

[5] Iseri L.T. (1984). Magnesium in coronary artery disease. Drugs.

[6] Li F.Y., Chaigne-Delalande B., Kanellopoulou C., Davis J.C., Matthews H.F., Douek D.C., Cohen J.I., Uzel G., Su H.C., Lenardo M.J. (2011). Second messenger role for Mg^2+^ revealed by human T-cell immunodeficiency. Nature.

[7] Alfrey A.C., Miller N.L. (1973). Bone Magnesium Pools in Uremia. J. Clin. Investig..

[8] De Baaij J.H., Hoenderop J.G., Bindels R.J. (2015). Magnesium in man: Implications for health and disease. Physiol. Rev..

[9] Ferré S., Hoenderop J.G., Bindels R.J. (2011). Insight into renal Mg^2+^ transporters. Curr. Opin. Nephrol. Hypertens..

[10] Giménez-Mascarell P., Schirrmacher C.E., Martínez-Cruz L.A., Müller D. (2018). Novel Aspects of Renal Magnesium Homeostasis. Front. Pediatr..

[11] Schäffers O.J.M., Hoenderop J.G.J., Bindels R.J.M., de Baaij J.H.F. (2018). The rise and fall of novel renal magnesium transporters. Am. J. Physiol. Ren. Physiol..

[12] Walder R.Y., Landau D., Meyer P., Shalev H., Tsolia M., Borochowitz Z., Boettger M.B., Beck G.E., Englehardt R.K., Carmi R. (2002). Mutation of TRPM6 causes familial hypomagnesemia with secondary hypocalcemia. Nat. Genet..

[13] Rude R.K., Gruber H.E. (2004). Magnesium deficiency and osteoporosis: Animal and human observations. J. Nutr. Biochem..

[14] Polok B., Escher P., Ambresin A., Chouery E., Bolay S., Meunier I., Nan F., Hamel C., Munier F.L., Thilo B. (2009). Mutations in CNNM4 cause recessive cone-rod dystrophy with amelogenesis imperfecta. Am. J. Hum. Genet..

[15] Wolf F.I., Trapani V. (2012). Magnesium and its transporters in cancer: A novel paradigm in tumour development. Clin. Sci..

[16] Luder H.U., Gerth-Kahlert C., Ostertag-Benzinger S., Schorderet D.F. (2013). Dental phenotype in Jalili syndrome due to a c.1312 dupC homozygous mutation in the CNNM4 gene. PLoS ONE.

[17] Arjona F.J., de Baaij J.H., Schlingmann K.P., Lameris A.L., van Wijk E., Flik G., Regele S., Korenke G.C., Neophytou B., Rust S. (2014). CNNM2 mutations cause impaired brain development and seizures in patients with hypomagnesemia. PLoS Genet..

[18] Funato Y., Yamazaki D., Mizukami S., Du L., Kikuchi K., Miki H. (2014). Membrane protein CNNM4-dependent Mg^2+^ efflux suppresses tumor progression. J. Clin. Investig..

[19] Yamazaki D., Miyata H., Funato Y., Fujihara Y., Ikawa M., Miki H. (2016). The Mg^2+^ transporter CNNM4 regulates sperm Ca^2+^ homeostasis and is essential for reproduction. J. Cell Sci..

[20] Wang C.Y., Shi J.D., Yang P., Kumar P.G., Li Q.Z., Run Q.G., Su Y.C., Scott H.S., Kao K.J., She J.X. (2003). Molecular cloning and characterization of a novel gene family of four ancient conserved domain proteins (ACDP). Gene.

[21] Ereño-Orbea J., Oyenarte I., Martínez-Cruz L.A. (2013). CBS domains: Ligand binding sites and conformational variability. Arch. Biochem. Biophys..

[22] Stuiver M., Lainez S., Will C., Terryn S., Günzel D., Debaix H., Sommer K., Kopplin K., Thumfart J., Kampik N.B. (2011). CNNM2, encoding a basolateral protein required for renal Mg^2+^ handling, is mutated in dominant hypomagnesemia. Am. J. Hum. Genet..

[23] Yamazaki D., Funato Y., Miura J., Sato S., Toyosawa S., Furutani K., Kurachi Y., Omori Y., Furukawa T., Tsuda T. (2013). Basolateral Mg^2+^ extrusion via CNNM4 mediates transcellular Mg^2+^ transport across epithelia: A mouse model. PLoS Genet..

[24] Hardy S., Uetani N., Wong N., Kostantin E., Labbé D.P., Bégin L.R., Mes-Masson A., Miranda-Saavedra D., Tremblay M.L. (2015). The protein tyrosine phosphatase PRL-2 interacts with the magnesium transporter CNNM3 to promote oncogenesis. Oncogene.

[25] Sponder G., Mastrototaro L., Kurth K., Merolle L., Zhang Z., Abdulhanan N., Smorodchenko A., Wolf K., Fleig A., Penner R. (2016). Human CNNM2 is not a Mg^2+^ transporter per se. Pflugers Arch..

[26] Kolisek M., Sponder G., Pilchova I., Cibulka M., Tatarkova Z., Werner T., Racay P. (2018). Magnesium Extravaganza: A critical compendium of current research into cellular Mg^2+^ transporters other than TRPM6/7. Rev. Physiol. Biochem. Pharmacol..

[27] De Baaij J.H., Stuiver M., Meij I.C., Lainez S., Kopplin K., Venselaar H., Müller D., Bindels R.J., Hoenderop J.G. (2012). Membrane topology and intracellular processing of cyclin M2 (CNNM2). J. Biol. Chem..

[28] Alderton A., Davies P., Illman K., Brown D.R. (2007). Ancient conserved domain protein-1 binds copper and modifies its retention in cells. J. Neurochem..

[29] Chandran U., Indu S., Kumar A.T., Devi A.N., Khan I., Srivastava D., Kumar P.G. (2016). Expression of Cnnm1 and Its Association with Stemness, Cell Cycle, and Differentiation in Spermatogenic Cells in Mouse Testis. Biol. Reprod..

[30] Wang C.Y., Yang P., Shi J.D., Purohit S., Guo D., An H., Gu J.G., Ling J., Dong Z., She J.X. (2004). Molecular cloning and characterization of the mouse Acdp gene family. BMC Genom..

[31] Chandran U., Laloraya M., Pradeep Kumar G. (2007). Identification of Testis-Expressed Cell Cycle Regulating Proteins with Special Reference to Meiosis. J. Endocrinol. Reprod..

[32] Voets T., Janssens A., Droogmans G., Nilius B. (2004). Outer pore architecture of a Ca^2+^-selective TRP channel. J. Biol. Chem..

[33] Glaudemans B., Knoers N.V., Hoenderop J.G., Bindels R.J. (2010). New molecular players facilitating Mg^2+^ reabsorption in the distal convoluted tubule. Kidney Int..

[34] Goytain A., Quamme G.A. (2005). Functional characterization of ACDP2 (ancient conserved domain protein) a divalent metal transporter. Physiol. Genom..

[35] Quamme G.A. (2010). Molecular identification of ancient and modern mammalian magnesium transporters. Am. J. Physiol. Cell Physiol..

[36] Won J., Kim J.H., Oh S.B. (2018). Molecular expression of Mg^2+^ regulator TRPM7 and CNNM4 in rat odontoblasts. Arch. Oral Biol..

[37] Quamme G., Biber J., Murer H. (1989). Sodium-phosphate cotransport in OK cells: Inhibition by PTH and “adaptation” to low phosphate. Am. J. Physiol..

[38] Ohi K., Hashimoto R., Ikeda M., Yamamori H., Yasuda Y., Fujimoto M., Umeda-Yano S., Fukunaga M., Fujino H., Watanabe Y. (2015). Glutamate Networks Implicate Cognitive Impairments in Schizophrenia: Genome-Wide Association Studies of 52 Cognitive Phenotypes. Schizophr Bull..

[39] Funato Y., Yamazaki D., Miki H. (2017). Renal function of cyclin M2 Mg^2+^ transporter maintains blood pressure. J. Hypertens..

[40] Kieboom B.C.T., Ligthart S., Dehghan A., Kurstjens S., de Baaij J.H.F., Franco O.H., Hofman A., Zietse R., Stricker B.H., Hoorn E.J. (2017). Serum magnesium and the risk of prediabetes: A population-based cohort study. Diabetologia.

[41] Lv W.Q., Zhang X., Zhang Q., He J.Y., Liu H.M., Xia X., Fan K., Zhao Q., Shi X.Z., Zhang W.D. (2017). Novel common variants associated with body mass index and coronary artery disease detected using a pleiotropic cFDR method. J. Mol. Cell Cardiol..

[42] Hirata Y., Funato Y., Takano Y., Miki H. (2014). Mg^2+^-dependent interactions of ATP with the cystathionine-β-synthase (CBS) domains of a magnesium transporter. J. Biol. Chem..

[43] Gulerez I., Funato Y., Wu H., Yang M., Kozlov G., Miki H., Gehring K. (2016). Phosphocysteine in the PRL-CNNM pathway mediates magnesium homeostasis. EMBO Rep..

[44] Zhang H., Kozlov G., Li X., Wu H., Gulerez I., Gehring K. (2017). PRL3 phosphatase active site is required for binding the putative magnesium transporter CNNM3. Sci. Rep..

[45] Giménez-Mascarell P., Oyenarte I., Hardy S., Breiderhoff T., Stuiver M., Kostantin E., Diercks T., Pey A.L., Ereño-Orbea J., Martínez-Chantar M.L. (2017). Structural Basis of the Oncogenic Interaction of Phosphatase PRL-1 with the Magnesium Transporter CNNM2. J. Biol. Chem..

[46] Kostantin E., Hardy S., Valinsky W.C., Kompatscher A., de Baaij J.H., Zolotarov Y., Landry M., Uetani N., Martínez-Cruz L.A., Hoenderop J.G. (2016). Inhibition of PRL-2·CNNM3 Protein Complex Formation Decreases Breast Cancer Proliferation and Tumor Growth. J. Biol. Chem..

[47] Parry D.A., Mighell A.J., El-Sayed W., Shore R.C., Jalili I.K., Dollfus H., Bloch-Zupan A., Carlos R., Carr I.M., Downey L.M. (2009). Mutations in CNNM4 cause Jalili syndrome, consisting of autosomal-recessive cone-rod dystrophy and amelogenesis imperfecta. Am. J. Hum. Genet..

[48] Cherkaoui Jaouad I., Lyahyai J., Guaoua S., El Alloussi M., Zrhidri A., Doubaj Y., Boulanouar A., Sefiani A. (2017). Novel splice site mutation in CNNM4 gene in a family with Jalili syndrome. Eur. J. Med. Genet..

[49] Coppieters F., Van Schil K., Bauwens M., Verdin H., De Jaegher A., Syx D., Sante T., Lefever S., Abdelmoula N.B., Depasse F. (2014). Identity-by descent -guided mutation analysis and exome sequencing in consanguineous families reveals unusual clinical and molecular findings in retinal dystrophy. Genet. Med..

[50] Rahimi-Aliabadi S., Daftarian N., Ahmadieh H., Emamalizadeh B., Jamshidi J., Tafakhori A., Ghaedi H., Noroozi R., Taghav S. (2016). A novel mutation and variable phenotypic expression in a large consanguineous pedigree with Jalili. Eye.

[51] Lopez Torres L.T., Schorderet D., Valmaggia C., Todorova M. (2015). A novel mutation in CNNM4 (G492C) associated with Jalili Syndrome. Acta Ophthalmol..

[52] Abu-Safieh L., Alrashed M., Anazi S., Alkuraya H., Khan A.O., Al-Owain M., Al-Zahrani J., Al-Abdi L., Hashem M., Al-Tarimi S. (2013). Autozygomeguided exome sequencing in retinal dystrophy patients reveals pathogenetic mutations and novel candidate disease genes. Genome Res..

[53] Prasad K., Geoffroy V., Vicaire S., Jost B., Dumas M., Le Gras S., Switala M., Gasse B., Laugel-Haushalter V., Paschaki M. (2016). A targeted next-generation sequencing assay for the molecular diagnosis of genetic disorders with orodental involvement. J. Med. Genet..

[54] Doucette L., Green J., Black C., Schwartzentruber J., Johnson G.J., Galutira D., Young T.L. (2013). Molecular genetics of achromatopsia in Newfoundland reveal genetic heterogeneity, founder effects and the first cases of Jalili syndrome in North America. Ophthalmic Genet..

[55] Topçu V., Alp M.Y., Alp C.K., Bakır A., Geylan D., Yılmazoğlu M.Ö. (2016). A new familial case of Jalili syndrome caused by a novel mutation in CNNM4. Ophthalmic Genet..

[56] Maia C.M.F., Machado R.A., Gil-da-Silva-Lopes V.L., Lustosa-Mendes E., Rim P.H.H., Dias V.O., Martelli D.R.B., Nasser L.S., Coletta R.D., Martelli-Júnior H. (2018). Report of two unrelated families with Jalili syndrome and a novel nonsense heterozygous mutation in CNNM4 gene. Eur. J. Med. Genet..

[57] Armitano J., Redder P., Guimarães V.A., Linder P. (2016). An Essential Factor for High Mg^2+^ Tolerance of Staphylococcus aureus. Front. Microbiol..

[58] Corral-Rodríguez M.A., Stuiver M., Abascal-Palacios G., Diercks T., Oyenarte I., Ereño-Orbea J., Ibáñez de Opakua A., Blanco F.J., Encinar J.A., Spiwok V. (2014). Nucleotide binding triggers a conformational change of the CBS module of the magnesium transporter CNNM2 from a twisted towards a flat structure. Biochem. J..

[59] Chen Y.S., Kozlov G., Fakih R., Funato Y., Miki H., Gehring K. (2018). The cyclic nucleotide-binding homology domain of the integral membrane protein CNNM mediates dimerization and is required for Mg^2+^ efflux activity. J. Biol. Chem..

[60] Kushwaha H.R., Singh A.K., Sopory S.K., Singla-Pareek S.L., Pareek A. (2009). Genome wide expression analysis of CBS domain containing proteins in *Arabidopsis thaliana* (L.) Heynh and *Oryza sativa* L. reveals their developmental and stress regulation. BMC Genom..

[61] Sinharoy S., Liu C., Breakspear A., Guan D., Shailes S., Nakashima J., Zhang S., Wen J., Torres-Jerez I., Oldroyd G. (2016). A *Medicago truncatula* Cystathionine Beta Synthase like domain-containing protein is required for rhizobial infection and symbiotic nitrogen fixation. Plant Physiol..

[62] Baykov A.A., Tuominen H.K., Lahti R. (2011). The CBS domain: A protein module with an emerging prominent role in regulation. ACS Chem. Biol..

[63] Bateman A. (1997). The structure of a domain common to archaebacteria and the homocystinuria disease protein. Tresarnds Biochem. Sci..

[64] Mahmood N.A.B.N., Biemans-Oldehinkel E., Poolman B. (2009). Engineering of Ion Sensing by the Cystathionine β-Synthase Module of the ABC Transporter OpuA. J. Biol. Chem..

[65] Zeng Q., Hong W., Tan Y.H. (1998). Mouse PRL-2 and PRL-3, two potentially prenylated protein tyrosine phosphatases homologous to PRL-1. Biochem. Biophys. Res. Commun..

[66] Lolicato M., Nardini M., Gazzarrini S., Möller S., Bertinetti D., Herberg F.W., Bolognesi M., Martin H., Fasolini M., Bertrand J.A. (2011). Tetramerization dynamics of C-terminal domain underlies isoform-specific cAMP gating in hyperpolarization-activated cyclic nucleotide-gated channels. J. Biol. Chem..

[67] Pessoa J., Fonseca F., Furini S., Morais-Cabral J.H. (2014). Determinants of ligand selectivity in a cyclic nucleotide-regulated potassium channel. J. Gen. Physiol..

[68] Brelidze T.I., Carlson A.E., Sankaran B., Zagotta W.N. (2012). Structure of the carboxy-terminal region of a KCNH channel. Nature.

[69] Feng N., Qi C., Hou Y.J., Zhang Y., Wang D.C., Li D.F. (2018). The C2′- and C3′-endo equilibrium for AMP molecules bound in the cystathionine-beta-synthase domain. Biochem. Biophys. Res. Commun..

[70] Bonifacino J.S. (2014). Adaptor proteins involved in polarized sorting. J. Cell Biol..

[71] González A., Rodriguez-Boulan E., Clathrin A.P.B. (2009). Key roles in basolateral trafficking through trans-endosomal routes. FEBS Lett..

[72] Traub L.M., Bonifacino J.S. (2013). Cargo recognition in clathrin-mediated endocytosis. Cold Spring Harb. Perspect. Biol..

[73] Scott J.W., Hawley S.A., Green K.A., Anis M., Stewart G., Scullion G.A., Norman D.G., Hardie D.G. (2004). CBS domains form energy sensing modules whose binding of adenosine ligands is disrupted by disease mutations. J. Clin. Investig..

[74] Shabb J.B., Corbin J.D. (1992). Cyclic nucleotide-binding domains in proteins having diverse functions. J. Biol. Chem..

[75] Tomita A., Zhang M., Jin F., Zhuang W., Takeda H., Maruyama T., Osawa M., Hashimoto K.I., Kawasaki H., Ito K. (2017). ATP-dependent modulation of MgtE in Mg^2+^ homeostasis. Nat. Commun..

[76] Meyer S., Savaresi S., Forster I.C., Dutzler R. (2007). Nucleotide recognition by the cytoplasmic domain of the human chloride transporter ClC-5. Nat. Struct. Mol. Biol..

[77] Julien S.G., Dubé N., Hardy S., Tremblay M.L. (2011). Inside the human cancer tyrosine phosphatome. Nat. Rev. Cancer.

[78] Hardy S., Wong N.N., Muller W.J., Park M., Tremblay M.L. (2010). Overexpression of the protein tyrosine phosphatase PRL-2 correlates with breast tumor formation and progression. Cancer Res..

[79] Kobayashi A., Mugford J.W., Krautzberger A.M., Naiman N., Liao J., McMahon A.P. (2014). Identification of a multipotent self-renewing stromal progenitor population during mammalian kidney organogenesis. Stem Cell Rep..

[80] Hattori M., Tanaka Y., Fukai S., Ishitani R., Nureki O. (2007). Crystal structure of the MgtE Mg^2+^ transporter. Nature.

[81] Hattori M., Iwase N., Furuya N., Tanaka Y., Tsukazaki T., Ishitani R., Maguire M.E., Ito K., Maturana A., Nureki O. (2009). Mg^(2+)^-dependent gating of bacterial MgtE channel underlies Mg^(2+)^ homeostasis. EMBO J..

[82] Sponder G., Svidova S., Schweigel M., Vormann J., Kolisek M. (2010). Splice-variant 1 of the ancient domain protein 2 (ACDP2) complements the magnesium-deficient growth phenotype of Salmonella enterica sv. typhimurium strain MM281. Magnes Res..

